# Comparative Antibacterial and Efflux Pump Inhibitory Activity of Isolated Nerolidol, Farnesol, and α-Bisabolol Sesquiterpenes and Their Liposomal Nanoformulations

**DOI:** 10.3390/molecules28227649

**Published:** 2023-11-17

**Authors:** Jorge Ederson Gonçalves Santana, Cícera Datiane de Morais Oliveira-Tintino, Gabriel Gonçalves Alencar, Gustavo Miguel Siqueira, Daniel Sampaio Alves, Talysson Felismino Moura, Saulo Relison Tintino, Irwin Rose Alencar de Menezes, João Pedro Viana Rodrigues, Vanessa Barbosa Pinheiro Gonçalves, Roberto Nicolete, Talha Bin Emran, Clara Mariana Gonçalves Lima, Sheikh F. Ahmad, Henrique Douglas Melo Coutinho, Teresinha Gonçalves da Silva

**Affiliations:** 1 Departamento de Antibióticos, Universidade Federal de Pernambuco (UFPE), Recife 50670-901, Brazil; edersantana22@hotmail.com (J.E.G.S.); teresinha.goncalves@ufpe.br (T.G.d.S.); 2 Departament of Biological Chemistry, Universidade Regional do Cariri (URCA), Crato 63105-010, Brazil; datianemorais@hotmail.com (C.D.d.M.O.-T.); gabriel.goncalves101@urca.br (G.G.A.); gustavo.miguelsiqueira@urca.br (G.M.S.); daniel.sampaio10@urca.br (D.S.A.); talysson.f.moura@urca.br (T.F.M.); saulorelison@gmail.com (S.R.T.); irwin.alencar@urca.br (I.R.A.d.M.); 3 Oswaldo Cruz Foundation (Fiocruz Ceará), Eusebio 61773-270, Brazil; jpedroviana@alu.ufc.br (J.P.V.R.); pinheiro.vanessaf@gmail.com (V.B.P.G.); rnicolete@gmail.com (R.N.); 4 Department of Pathology and Laboratory Medicine, Warren Alpert Medical School, Brown University, Providence, RI 02912, USA; talhabmb@bgctub.ac.bd; 5 Legorreta Cancer Center, Brown University, Providence, RI 02912, USA; 6 Department of Pharmacy, Faculty of Allied Health Sciences, Daffodil International University, Dhaka 1207, Bangladesh; 7 Department of Food Science, Federal University of Lavras, Lavras 37203-202, Brazil; 8 Department of Pharmacology and Toxicology, College of Pharmacy, King Saud University, Riyadh 11451, Saudi Arabia

**Keywords:** efflux pump, fluorescence, liposome, nanoformulation, sesquiterpenes, *Staphylococcus aureus*

## Abstract

The efflux systems are considered important mechanisms of bacterial resistance due to their ability to extrude various antibiotics. Several naturally occurring compounds, such as sesquiterpenes, have demonstrated antibacterial activity and the ability to inhibit efflux pumps in resistant strains. Therefore, the objective of this research was to analyze the antibacterial and inhibitory activity of the efflux systems NorA, Tet(K), MsrA, and MepA by sesquiterpenes nerolidol, farnesol, and α-bisabolol, used either individually or in liposomal nanoformulation, against multi-resistant *Staphylococcus aureus* strains. The methodology consisted of in vitro testing of the ability of sesquiterpenes to reduce the Minimum Inhibitory Concentration (MIC) and enhance the action of antibiotics and ethidium bromide (EtBr) in broth microdilution assays. The following strains were used: *S. aureus* 1199B carrying the NorA efflux pump, resistant to norfloxacin; IS-58 strain carrying Tet(K), resistant to tetracyclines; RN4220 carrying MsrA, conferring resistance to erythromycin. For the EtBr fluorescence measurement test, K2068 carrying MepA was used. It was observed the individual sesquiterpenes exhibited better antibacterial activity as well as efflux pump inhibition. Farnesol showed the lowest MIC of 16.5 µg/mL against the *S. aureus* RN4220 strain. Isolated nerolidol stood out for reducing the MIC of EtBr to 5 µg/mL in the 1199B strain, yielding better results than the positive control CCCP, indicating strong evidence of NorA inhibition. The liposome formulations did not show promising results, except for liposome/farnesol, which reduced the MIC of EtBr against 1199B and RN4220. Further research is needed to evaluate the mechanisms of action involved in the inhibition of resistance mechanisms by the tested compounds.

## 1. Introduction

The indiscriminate use of antibiotics has influenced the alarming levels of bacterial resistance to multiple drugs. Bacterial resistance to antibiotics is a natural, evolutionary, and adaptive phenomenon of these microorganisms, causing inactivation or reducing the action of antibiotics and biocides. Among the existing resistance mechanisms, active efflux systems stand out, which reduce the intracellular concentration of the antibiotic in the bacterial cell [1,2,3]

Efflux pumps are considered one of the most important mechanisms of bacterial resistance due to their broad range of substrates. They can be found in Gram-positive and Gram-negative bacteria, facilitating the extrusion of almost all existing classes of conventional antibiotics [4,5,6,7,8,9].

Research on efflux pumps is growing, aiming to develop or enhance effective drugs. In this perspective, many studies have evaluated medicinal plants that show high potential for inhibiting bacterial infections [10]. Many bioactive compounds present in medicinal plants demonstrate direct antimicrobial activity, synergistic action, and potentiation of drugs, as well as inhibition of bacterial resistance mechanisms. Among the studied classes, sesquiterpenes are active metabolites of essential oils from medicinal plants that possess important antimicrobial characteristics [11,12].

Studies have shown sesquiterpenes exhibit activity in inhibiting efflux pumps in resistant strains of *Staphylococcus aureus*. Furthermore, formulation and encapsulation studies have demonstrated liposomal nanoformulations can enhance absorption, improve distribution, and prolong the plasma half-life of compounds such as sesquiterpenes, which have limitations in their pharmacological potential due to their low solubility in biological fluids [13,14,15,16].

Liposomes are artificial vesicles composed of one or more concentric phospholipid bilayers. They are formed from phospholipids and cholesterol, which are biocompatible and non-toxic materials. These phospholipids have a hydrophilic head and a hydrophobic tail made of fatty acids, providing compartments of different polarities and compatibility for encapsulating hydrophilic or hydrophobic compounds. They have the ability to trap lipophilic agents in the lipid membrane and hydrophilic compounds in the aqueous core. The physicochemical properties of liposomes, such as permeability, membrane fluidity, charge density, determine the interaction of liposomes with the body’s targets after systemic administration, making them an efficient drug carrier system [17,18,19].

Research has shown the significant bioactivity of naturally sourced compounds encapsulated in liposomes. Among these biological activities, one can mention antibacterial action [20], antioxidant [21,22,23], antitumoral [24,25], and protection against neurodegenerative diseases [26]. Sesquiterpenes are organic compounds with lipophilic characteristics. Many substances, such as proteins, lipid-polymer conjugates, and fats, are frequently used as vehicles for lipophilic substances. The use of nanoformulation technology facilitates the delivery of sesquiterpenes without altering their organoleptic characteristics and physicochemical properties [27,28].

Innovations using vesicular carriers are on the rise due to their proven effectiveness in drug delivery, enhancing the bioactivity of compounds for topical or systemic action [29]. Various vesicular carrier systems have demonstrated in vitro antimicrobial action, leading to the optimization of the drug [30].

Currently, research into new targets for antibiotic therapy is on the rise. In this regard, the search for different strategies to inhibit efflux pumps is essential to restore the effectiveness of antibiotics. Therefore, the discovery of natural compounds that can act as antibiotic adjuvants or inhibit resistance mechanisms becomes relevant. Furthermore, the liposomal encapsulation of bioactive molecules has shown promise in antimicrobial treatment because liposomes enhance the delivery and distribution of the drug within biological systems [20].

In light of this, the present study aims to analyze the inhibitory activity of the efflux systems NorA, Tet(K), MsrA, and MepA by sesquiterpenes nerolidol, farnesol, and α-bisabolol, used individually and in liposomal nanoformulation, against multi-resistant *S. aureus* strains.

## 2. Results and Discussion

### 2.1. Physical-Chemical Profile of Liposomes

The physical-chemical characterization of the nanoformulations consisted of determining the average size of the liposomes, intensity, Zeta potential, concentration, polydispersity index (PDI), and encapsulation efficiency (Table 1).

The nanoformulations presented average sizes of 218.9 nm, 241.8 nm, 201.4 nm, and 183.5 nm, respectively, for the control liposomes and those containing nerolidol, farnesol, and α-bisabolol. The average size of nanoparticles is a parameter that can influence their biological activity. Different particle sizes can exhibit distinct degrees of biodistribution, absorption, and therapeutic efficacy in different targets. Approaching liposome particle sizes around 100 nm, as seen in the α-bisabolol formulation, is particularly relevant because this size is associated with enhanced biological activity. This implies the formulation can be more effective in delivering its active compounds, leading to superior therapeutic outcomes. However, it is important to emphasize the final biological activity is also influenced by factors such as liposome composition, surface charge, morphology, and release properties of the encapsulated active compound [31,32].

Intensity is a widely used technique for determining the concentration and size of particles in suspension. Intensity is related to the number of particles in the sample and the encapsulation efficiency of the encapsulated sesquiterpenes. This parameter is used as an indirect measure to estimate the amount of encapsulated agent relative to the total quantity of particles present [33]. The present study showed satisfactory signal intensity, which was 2.5 a.u., 3 a.u., 5 a.u., and 5 a.u., respectively.

The Zeta potential, ranging from −24.1 mV to 28.2 mV in the formulations, serves as an indicator of particle charge and colloidal stability. The Zeta potential is a measure of the electric charge of nanoparticles and is indicative of their colloidal stability. This value provides information about the electrostatic repulsion between particles, the tendency of aggregation, and the interaction with cells and tissues. The potential is determined by the potential difference between the surface of the particles and the surrounding dispersing liquid. It is influenced by various factors such as ionic strength, suspension composition, pH, and interfacial interactions [34,35]. Values further from zero indicate more suspension stability. Therefore, the liposomes/sesquiterpenes studied show significant Zeta potential values, which are −18.8 mV, −24.1 mV, −13 mV, and 28.2 mV. Significant Zeta potential values, as observed in the nerolidol and α-bisabolol formulations, suggest good colloidal stability.

Concentration refers to the quantity of nanoparticles present per ml of the analyzed sample. For instance, the nanoformulation containing nerolidol has a concentration of 6.82 × 10^8^ particles/mL, the farnesol formulation contains 4.42 × 10^8^ particles/mL, and the nanoformulation with α-bisabolol contains 4.02 particles/mL. In addition to implications related to safety and efficacy, nanoparticle concentration can also impact the physical and chemical properties of the particles. For example, the colloidal stability of nanoparticles can be influenced by the concentration. A study conducted by Hufschmid et al. [36] investigated the stability of iron oxide nanoparticles at different concentrations. The results revealed high concentrations of nanoparticles led to a higher rate of agglomeration and sedimentation, which could compromise the colloidal stability of the particles.

The concentration of nanoparticles in the solution can influence the dosage and therapeutic efficacy. Although higher concentrations of nanoparticles result in greater bioactivity and faster biodisponibility, it can also potentiate their toxicity [37]. High concentrations of nanoparticles can accumulate in tissues and organs, leading to oxidative stress, cellular damage, and consequently, adverse effects. Therefore, it is necessary to find the ideal concentration for pharmacological action [38].

The Polydispersity Index is a measure of particle size uniformity within the sample. PDI values range from 0.50 (control group) to 0.92 (farnesol formulation). A PDI close to 1 indicates particles have similar sizes, whereas a higher PDI suggests a broader size distribution. The farnesol formulation exhibits a higher PDI, which may indicate greater variation in particle size. The elevated PDI observed in some liposome formulations (up to 0.92) suggests a broader size distribution of suspended particles. However, it is worth highlighting the suitability of the microfluidic technique employed in this study. Microfluidics is renowned for its precision in liposome formation, allowing for precise control of particle size and uniformity. The high mixing efficiency and shear within microchannels result in consistently sized liposomes. Microfluidics can be more lipid-efficient compared to other methods, thereby reducing costs, and optimizing formulation [39,40].

The encapsulation efficiency refers to the amount of sesquiterpene encapsulated In the lipid nanoparticle. Higher values indicate greater encapsulation efficiency. For example, the liposomes studied here showed an encapsulation efficiency of 85%, 79%, and 87%, respectively, for nerolidol, farnesol, and bisabolol, indicating a high percentage of the compound inside the liposome.

A study conducted by Minelli et al. [41] investigated the efficacy of solid lipid nanoparticles carrying farnesol in inhibiting the growth of colon cancer cells. The results showed farnesol-loaded nanoparticles exhibited a higher internalization rate in cancer cells and induced greater apoptosis compared to free farnesol. Additionally, the nanoparticles demonstrated lower toxicity to healthy cells. These studies highlight the importance of nanoparticles in enhancing the delivery efficiency of sesquiterpenes and improving their therapeutic activities. Nanoparticles allow for the protection of sesquiterpenes against degradation and enable their controlled release at the target site. Moreover, nanoparticle formulation can enhance the water solubility of sesquiterpenes, allowing for more effective administration [42].

### 2.2. Antibacterial Activity and Efflux Pump Inhibition Assessed through MIC Reduction

The isolated sesquiterpenes showed direct antibacterial activity, with nerolidol having MIC values of 32 µg/mL and 128 µg/mL against IS-58 and RN4220 strains, respectively. Farnesol presented MIC values of 25.4 µg/mL, 32 µg/mL, and 16 µg/mL against 1199B, IS-58, and RN4220 strains, respectively. α-bisabolol exhibited MIC values of 128 µg/mL, 64 µg/mL, and 161.3 µg/mL against 1199B, IS-58, and RN4220 strains, respectively (Table 2).

Given sesquiterpenes are organic compounds with lipophilic characteristics, they were incorporated into the lipophilic layer of the liposomes, becoming interspersed within their membrane. With this, the sesquiterpene can be gradually released upon contact with the bacterial membrane or the surrounding environment. The non-promising results observed with the liposome/sesquiterpene complex may have occurred due to the difference between the lipids present in the liposome and the phospholipids in the bacterial membrane of *S. aureus* [43,44].

**Figure 1:**
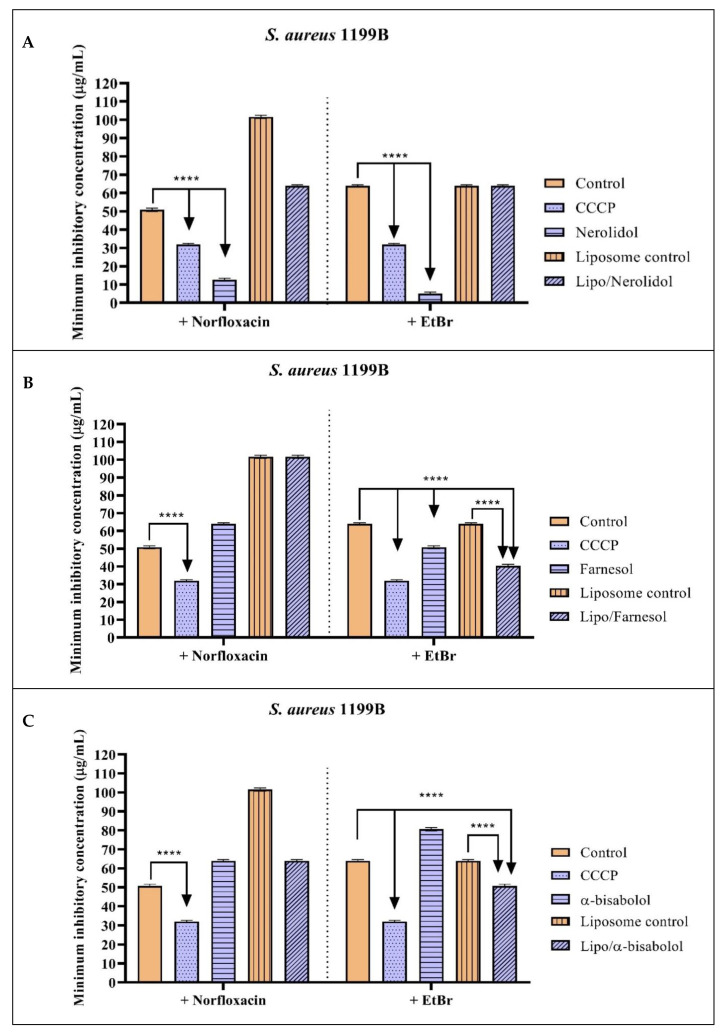
Evaluation of the NorA efflux pump inhibitory activity by nerolidol (**A**), farnesol (**B**), and α-bisabolol (**C**) sesquiterpenes isolated and encapsulated in liposomes, against the *S. aures* 1199B strain. Associated with norfloxacin and ethidium bromide. Two-way ANOVA followed by Bonferroni post hoc. CCCP = carbonyl cyanide 3-chlorophenylhydrazone; EtBr = ethidium bromide; **** = *p* < 0.0001 vs. control.

Figure 1 and Table 3 show the inhibitory action results of the sesquiterpenes nerolidol, farnesol, and α-bisabolol against *S. aureus* 1199B strains carrying the NorA efflux pump. In Figure 1A, it can be observed the combination of nerolidol with norfloxacin significantly reduced the MIC of this antibiotic from 50.6 µg/mL to 12.7 µg/mL, compared to the antibiotic control alone, indicating potentiation of the antibacterial activity. The combination of nerolidol with EtBr also reduced the EtBr MIC from 64 µg/mL to 5 µg/mL. Both in combination with the antibiotic and in combination with EtBr, nerolidol showed significantly better results than the positive control CCCP. However, nerolidol encapsulated in a liposomal nanoformulation did not show a potentiating effect on norfloxacin or EtBr.

Isolated or encapsulated farnesol, when associated with norfloxacin, did not show significant results in reducing the MIC. However, when associated with EtBr, both isolated and encapsulated farnesol reduced the EtBr MIC to 50 µg/mL and 40.3 µg/mL, respectively, compared to the control (64 µg/mL) (Figure 1B).

The α-bisabolol in association with norfloxacin did not potentiate the antibacterial action of the antibiotic. When associated with EtBr, isolated α-bisabolol did not have a synergistic effect. However, α-bisabolol in the liposomal nanoformulation showed a synergistic effect with a reduced MIC of 50.8 µg/mL, compared to the control of 64 µg/mL (Figure 1C). These results indicate the isolated sesquiterpenes nerolidol and farnesol possibly act on the inhibition of NorA, while the encapsulated forms of farnesol and α-bisabolol may act on the inhibition of NorA.

**Figure 2:**
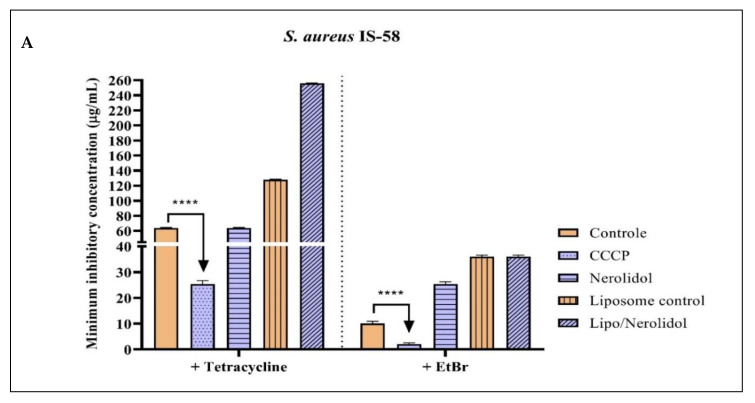

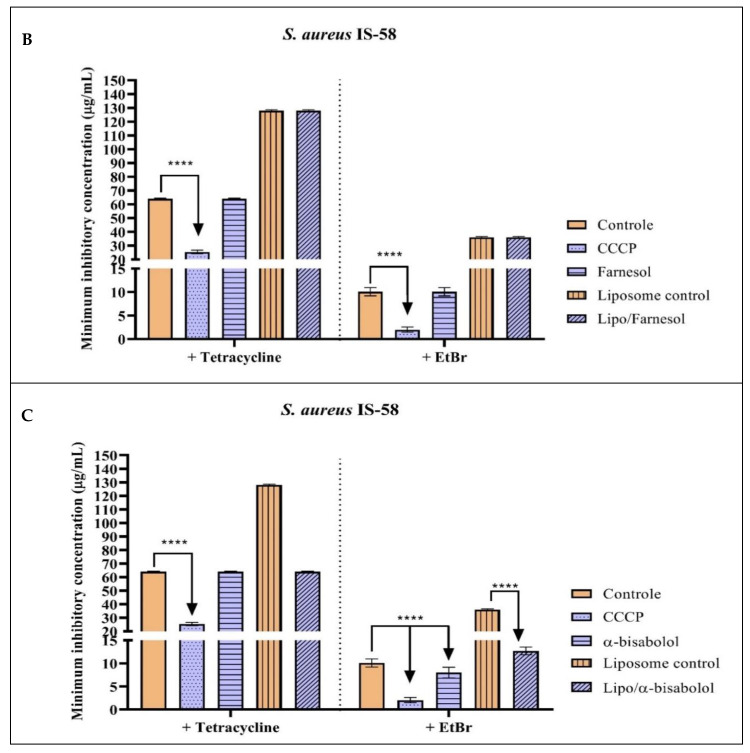
Evaluation of the Tet(K) efflux pump inhibitory activity by the nerolidol (**A**), farnesol (**B**), and α-bisabolol (**C**) sesquiterpenes isolated and encapsulated in liposomes, against the *S. aures* IS-58 strain. Associated with norfloxacin and ethidium bromide. Two-way ANOVA followed by Bonferroni post hoc. CCCP = Carbonyl cyanide 3-chlorophenylhydrazone; EtBr = ethidium bromide; **** = *p* < 0.0001 vs. control.

Figure 2 shows the results of the action of the sesquiterpenes nerolidol, farnesol, and α-bisabolol against the *S. aureus* IS-58 strain carrying the Tet(K) efflux pump. Among all substances tested, only α-bisabolol exhibited a potentiating effect, reducing the EtBr MIC to 8 µg/mL. This result indicates that α-bisabolol may inhibit the Tet(K) efflux mechanism in the *S. aureus* IS-58 strain (Table 4).

**Figure 3:**
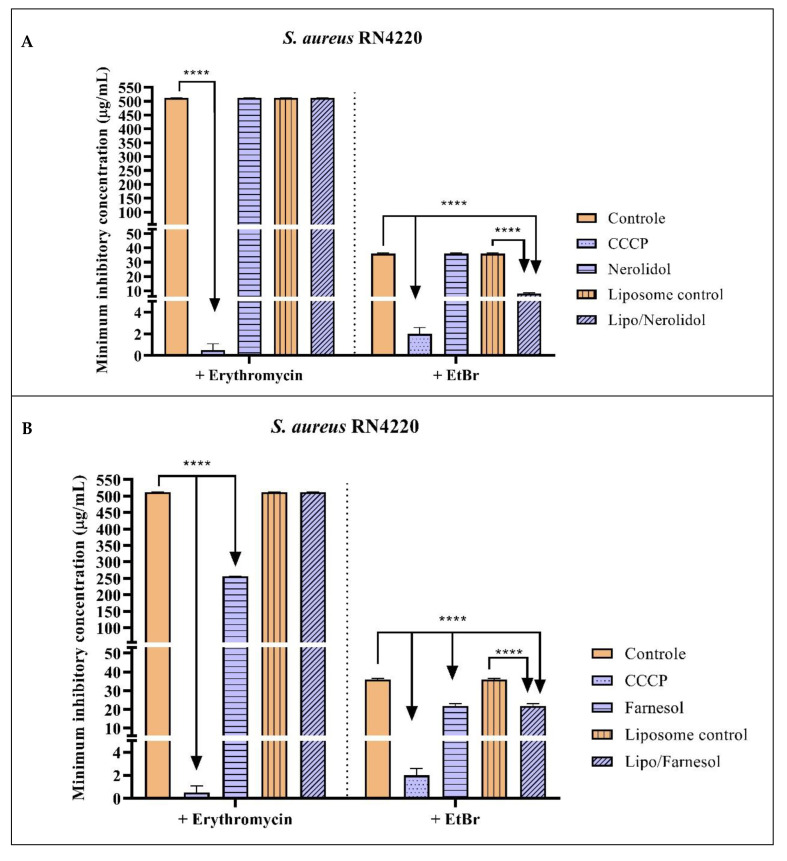

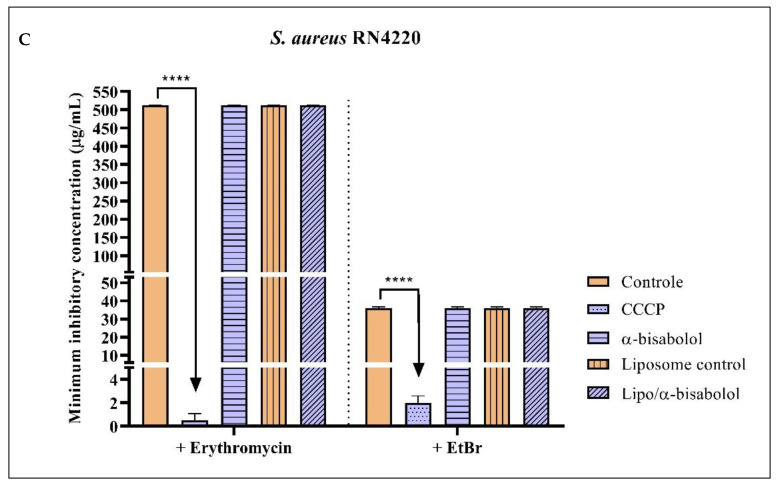
Evaluation of the MsrA efflux pump inhibitory activity by the nerolidol (**A**), farnesol (**B**), and α-bisabolol (**C**) sesquiterpenes isolated and encapsulated in liposomes, against the *S. aures* RN4220 strain. Associated with erythromycin and ethidium bromide. Two-way ANOVA followed by Bonferroni post hoc. CCCP = Carbonyl cyanide 3-chlorophenylhydrazone; EtBr = ethidium bromide; **** = *p* < 0.0001 vs. control.

Against the *S. aureus* strain expressing the MsrA efflux system, only farnesol showed significant effects when associated with erythromycin, reducing the erythromycin MIC to 256 µg/mL. In association with EtBr, encapsulated nerolidol and isolated and encapsulated farnesol showed significant effects in reducing the MIC to 8 µg/mL, 21 µg/mL, and 21.8 µg/mL, respectively, indicating the occurrence of MsrA efflux pump inhibition (Figure 3 and Table 5).

The reduction of specific antibiotics’ MIC and EtBr by sesquiterpenes is indicative of efflux pump inhibition or potentialization of antibiotic action [45,46,47,48,49,50,51,52,53]. Secondary metabolites are chemical compounds produced by plants as defense mechanisms against pathogens such as fungi and bacteria, as well as herbivorous animals. They can be useful in treating diseases and infections, exhibiting various proven bioactivities such as antioxidant, antidiabetic, antiproliferative, anti-inflammatory, and antimicrobial actions [54].

According to Alsheikh et al. [55], phytochemicals can exert antimicrobial action through mechanisms distinct from conventional antibiotics, such as inhibiting cell wall synthesis and interfering with bacterial physiology by reducing membrane potential and ATP synthesis. Additionally, they can modulate bacterial susceptibility to antibiotics.

Components of essential oils can act on efflux pumps, restoring the effectiveness of some antibiotics that are targets of resistance mechanisms. Sesquiterpenes exhibit broad antibacterial activity related to their lipophilic characteristics [56]. Oliveira et al. [57] emphasize that sesquiterpenes nerolidol, farnesol, and α-bisabolol have the potential to enhance the activity of conventional antimicrobials, such as gentamicin, oxacillin, and methicillin. Farnesol can potentiate the effect of conventional antimicrobials against *S. aureus* RN4220 strains that produce the MsrA efflux mechanism. In the study, researchers associated the sesquiterpene with fusidic acid, demonstrating the potentiation of this effect on the MIC.

According to Cruz et al. [58], α-bisabolol exhibited potentiating activity against antimicrobials in the presence of *S. aureus* strains expressing the Tet(K) and NorA efflux systems. In their studies, Moura et al. [11] demonstrated nerolidol is an effective sesquiterpene in infection treatment caused by multidrug-resistant bacteria.

In addition to the antimicrobial effects of sesquiterpenes on multidrug-resistant strains, studies reveal liposomal nanoformulations can enhance the therapeutic action of antimicrobials. The encapsulation of farnesol in liposomes resulted in significantly increased antifungal activity against strains of *Candida albicans*, *C. tropicalis*, and *C. krusei*, leading to a considerable reduction in IC50 [59].

Several in vivo and in vitro studies confirm the effectiveness of encapsulating compounds in liposomes, enhancing the antibacterial and anticancer action of these compounds [60,61,62,63]. These results are consistent with the data presented for farnesol in liposomes against the 1199B and RN4220 strains, where there was a reduction in the MIC of EtBr.

### 2.3. Evaluation of Efflux Pump Inhibition by Fluorescence Emission

**Figure 4:**
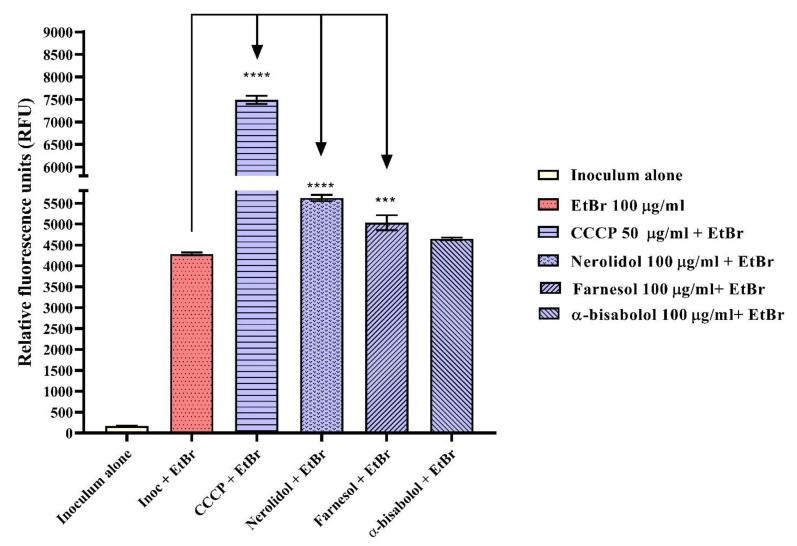
Evaluation of MepA efflux pump inhibition by measuring fluorescence emission in *S. aureus* K2068 strain, treated with nerolidol, farnesol, and α-bisabolol at 100 µg/mL. EtBr = ethidium bromide; Inoc = inoculum; **** = *p* < 0.0001 vs. inoc + EtBr; *** = *p* < 0.001 vs. inoc + EtBr.

When measuring fluorescence emission, it was observed nerolidol at 100 µg/mL and farnesol at 100 µg/mL increased fluorescence emission compared to the negative control, which consisted of inoculum plus EtBr. This increase was represented by 31.3% and 17.5%, respectively. The same result was observed with the efflux pump inhibitor CCCP, indicating the reproducibility of the experiment (Figure 4). The average increase in fluorescence suggested the possible inhibition of the MepA efflux pump, considering that inhibition of EtBr efflux led to an increase in its intracellular concentration and, consequently, enhanced the fluorescence of the sample [64,65,66].

## 3. Materials and Methods

### 3.1. Substances Used in Research

The sesquiterpenes nerolidol (C_15_H_26_O), farnesol (C_15_H_26_O), and α-bisabolol (C_15_H_26_) were used. Carbonyl cyanide m-chlorophenyl-hydrazone (CCCP) was used as the standard efflux pump inhibitor for positive control. The DNA intercalating agent used was ethidium bromide (EtBr). Specific antibiotics were used as substrates for each bacterial efflux pump: norfloxacin for the *S. aureus* 1199B strain carrying NorA; tetracycline for the *S. aureus* IS-58 strain carrying Tet(K); and erythromycin for the *S. aureus* RN4220 strain carrying the MsrA protein. The culture media used were solid medium Heart Infusion Agar (HIA, Difco, Forn El Chebbak, Lebanon) and liquid medium Brain Heart Infusion (BHI). All products were purchased from Sigma-Aldrich Chemical Co. (St. Louis, MO, USA).

### 3.2. Synthesis of Liposomal Nanoformulations

Initially, a highly concentrated solution of 50 mg/mL of nerolidol, farnesol, and α-bisabolol was prepared for encapsulation. To create the organic phase that constituted the liposomal nanoparticles, a lipid solution was prepared, consisting of 1,2-dipalmitoyl-sn-glycero-3-phosphocholine (DPPC), cholesterol (CHOL), and distearoylphosphatidylcholine (DSPC) in a ratio of DPPC:CHOL:DSPC at 52:45:3 (mol/mol) to achieve a final lipid concentration of 35 mM. The microfluidics technique was employed using the NanoAssemblr Benchtop equipment (Precision Nanosystems^TM^, Vancouver, BC, Cananda), For the nanoparticle fabrication. To optimize encapsulation efficiency, the following factors were used: a flow rate ratio of 2:1 and a total flow ratio of 12 mL/min for the left and right syringes, respectively. In the left syringe, the aqueous phase containing the diluted sesquiterpene solution was added until a total volume of 3 mL was reached. In the right syringe, the lipid solution was added with a total volume of 1 mL. For the preparation of control nanoformulations, or liposome controls, only phosphate-buffered saline (PBS) was added to the left syringe (aqueous solution). After the nanoparticle preparation, the resulting formulation was placed in Amicon^®^ Ultra-15 3000 MWCO (Merck, Darmstadt, Germany) and centrifuged at 3000 rpm, 20 °C for 30 min, to remove the residual solvent used in lipid solubilization. The washing was performed with PBS buffer at pH 7.2. Finally, the formulations were stored in a refrigerated environment at 3 °C to 8 °C [59].

### 3.3. Microorganisms Used in the Assays

The following strains were used: *S. aureus* 1199B, resistant to hydrophilic fluoroquinolones via the NorA efflux protein; *S. aureus* IS-58, containing the PT181 plasmid carrying the Tet(K) gene that extrudes tetracyclines; *S. aureus* RN4220, carrying the pUL5054 plasmid that carries the gene for the MsrA protein which effluxes macrolides (Table 6). All strains were maintained on HIA medium at 4 °C and in glycerol in a freezer at −80 °C. The resistance gene-carrying strains were maintained in culture medium under subinhibitory antibiotic conditions to induce gene expression.

### 3.4. Antibacterial Activity Evaluated by Measuring the Minimum Inhibitory Concentration (MIC)

This test consisted of determining the minimum inhibitory concentration (MIC) of sesquiterpenes capable of inhibiting the growth of *S. aureus* strains 1199B, IS-58, and RN4220. The bacterial inoculum of the three strains was prepared in sterile saline solution, corresponding to a McFarland scale of 0.5, which corresponded to 1.5 × 10^8^ Colony-Forming Units. Then, distribution media were prepared in Eppendorf tubes containing 900 µL of Brain Heart Infusion (BHI) culture medium and 100 µL of the inoculum. A total of 100 µL of the tube contents were transferred to a 96-well microdilution plate. Subsequently, a serial dilution (1:1) was performed with 100 µL of either isolated or encapsulated nerolidol, farnesol, or α-bisabolol sesquiterpenes. The microdilution was carried out until the penultimate well, leaving the last well as a growth control. The final concentrations of each sesquiterpene ranged from 512 µg/mL to 0.5 µg/mL. The plates were incubated in a bacteriological incubator for 24 h at 37 °C. The experiments were performed in triplicate. The reading was performed by adding 20 µL of resazurin (7-hydroxy-3H-phenoxazin-3-one 10-oxide), observing the change in color in each well. Blue coloration indicated the absence of bacterial growth, while a color change to red indicated bacterial growth [67,68].

### 3.5. Evaluation of the Inhibition of Efflux Pumps NorA, Tet(K), and MsrA

The inhibition of efflux pumps was verified by the reduction of MIC of antibiotics and EtBr against *S. aureus* strains 1199B, IS-58, and RN4220. Bacterial inocula were prepared as described in the previous section. Test solutions were prepared in Eppendorf tubes containing 200 µL of inoculum, isolated or encapsulated sesquiterpene at subinhibitory concentration (MIC/8), and BHI culture medium, resulting in a final volume of 2 mL. The control solution contained only the inoculum and culture medium. Subsequently, the solutions were transferred to 96-well microtiter plates, with the addition of 100 µL of the content in each well. Then, 100 µL of norfloxacin, tetracycline, erythromycin, or EtBr antibiotics were serially diluted (1:1) until the penultimate well, resulting in concentrations ranging from 512 µg/mL to 0.5 µg/mL. The negative control contained only the antibiotic or EtBr alone. The positive control consisted of CCCP. The reading was performed as described in the previous section. The MIC was defined as the lowest concentration at which there was no bacterial growth in the well, characterized by the blue coloration of resazurin [67,68].

### 3.6. Efflux Pumps Inhibition Evaluated by the Increased Fluorescence Emission of EtBr

The strain *S. aureus* K2068 was seeded on a solid HIA culture medium and incubated in a bacteriological incubator at 37 °C for 24 h before conducting the experiments. The inoculum was prepared until obtaining 1.5 × 10^8^ colony forming units (CFU), corresponding to the 0.5 value on the McFarland scale. The inoculum was prepared in PBS. For the test, sesquiterpenes were selected as they showed the best results in microdilution assays. Test solutions were prepared containing the K2068 inoculum and the sesquiterpenes nerolidol, farnesol, and α-bisabolol, all at 100 µg/mL. The positive control used was CCCP at 50 µg/mL. PBS buffer was added to each solution to reach a final volume of 1 mL. The solutions were incubated for 1 h and 30 min. Then, EtBr (ethidium bromide) at 100 µg/mL was added to all solutions except the inoculum alone group, which served as the growth control. The solutions were incubated for an additional 1 h. Subsequently, the solutions were centrifuged at 10,000 rpm for 2 min and washed with PBS to remove all EtBr and medium substances. The supernatant was discarded, and the resulting pellet was dissolved in PBS. The sample containing the dissolved pellet was distributed into microplates. The reading was performed using Cytation 1, BioTek^®^ (Winooski, VT, USA) fluorescence microplate reader and Gen5™ 3.22 Software with excitation at 530 nm and emission wavelength at 590 nm. The reading was taken for the following groups: inoculum alone (growth control), inoculum + EtBr (negative control), inoculum + EtBr + CCCP (positive control), inoculum + EtBr + nerolidol 100 µg/mL, inoculum + EtBr + farnesol 100 µg/mL, and inoculum + EtBr + α-bisabolol 100 µg/mL. The assay was performed in triplicate, and the results were compared to the negative control group, EtBr [12].

### 3.7. Statistical Analysis

The assays were performed in triplicate. In microbiological tests, descriptive statistics were used to calculate the geometric mean and standard deviation, and the results were compared using Two-way ANOVA, followed by the Bonferroni post hoc test. Analysis of the fluorimetry assay and other tests was carried out using One-way ANOVA, followed by the Dunnett test. Results were considered significant when *p* < 0.05. GraphPad Prism 5.0 software was used.

## 4. Conclusions

Therefore, when isolated, nerolidol exhibited direct antibacterial activity against *S. aureus* IS-58 and RN4220 strains. Isolated farnesol and α-bisabolol showed direct antibacterial activity against *S. aureus* 1199B, IS-58, and RN4220. However, the liposomal formulation of these compounds did not show direct efficacy against *S. aureus* strains. In terms of efflux pump inhibition, these compounds demonstrated effectiveness. Liposome/farnesol and liposome/α -bisabolol acted as potential inhibitors of NorA present in *S. aureus* 1199B. Liposome/nerolidol and liposome/farnesol acted as potential inhibitors of MsrA in the *S. aureus* RN4220 strain. The isolated sesquiterpenes showed significant action: in their isolated form, nerolidol and farnesol acted as putative inhibitors of NorA and MepA; α-bisabolol acted as a putative inhibitor of Tet(K), and isolated farnesol acted as inhibitor of MsrA and MepA. Among all the substances tested, isolated nerolidol stood out for its potent inhibition of NorA, being even more effective than CCCP. Further studies are necessary to describe the molecular targets involved in these mechanisms.

## Figures and Tables

**Table 1:** Physical-chemical characteristics of liposomal nanoformulations control and containing nerolidol, farnesol, and α-bisabolol.

	Liposome Control	Liposome/ Nerolidol	Liposome/ Farnesol	Liposome/ α-Bisabolol
Size	218.9 nm ± 45.1	241.8 nm ± 73.1	201.4 nm ± 66.6	183.5 nm ± 58
Intensity	2.5 a.u.	3 a.u.	5 a.u.	5 a.u.
Zeta potential	−18.8 mV	−24.1 mV	−13 mV	28.2 mV
pH	7.4	7.4	7.4	7.4
Concentration	6.99 × 10^8^ particles/mL	6.82 × 10^8^ particles/mL	4.42 × 10^8^ particles/mL	4.02 × 10^8^ particles/mL
Polydispersity index (PDI)	0.50	0.61	0.92	0.755
Encapsulation efficiency	-	85.4%	79%	87%

**Table 2:** The minimum inhibitory concentration of isolated and encapsulated nerolidol, farnesol, and α-bisabolol against *S. aureus* strains 1199B, IS-58, and RN4220. Concentrations in (µg/mL).

Compound	1199B	IS-58	RN4220
Nerolidol	1024 µg/mL ± 0.5	32 µg/mL ± 0.5 *	128 µg/mL ± 0.5 *
Farnesol	25.4 µg/mL ± 0.8 *	32 µg/mL ± 0.5 *	16 µg/mL ± 0.5 *
α-bisabolol	128 µg/mL ± 0.5 *	64 µg/mL ± 0.5 *	161.3 µg/mL ± 0.8 *
Liposome/Nerolidol	≥1024 µg/mL ± 0.5	≥1024 µg/mL ± 0.5	≥1024 µg/mL ± 0.5
Liposome/Farnesol	≥1024 µg/mL ± 0.5	≥1024 µg/mL ± 0.5	≥1024 µg/mL ± 0.5
Liposome/α-bisabolol	≥1024 µg/mL ± 0.5	≥1024 µg/mL ± 0.5	≥1024 µg/mL ± 0.5

* Clinically relevant antibacterial activity.

**Table 3:** Minimum inhibitory concentration of nerolidol, farnesol, and α-bisabolol sesquiterpenes, whether isolated or encapsulated in liposomes, against the *S. aureus* 1199B strain, associated with norfloxacin and ethidium bromide. * Statistically significant compared to the norfloxacin or EtBr control.

Group with Antibiotic	MIC	Group with EtBr	MIC
Norfloxacin	50.8 µg/mL ± 0.8	EtBr	64 µg/mL ± 0.5
Norfloxacin + CCCP	32 µg/mL ± 0.5 *	EtBr + CCCP	32 µg/mL ± 0.5 *
Norfloxacin + Nerolidol	12.7 µg/mL ± 0.8 *	EtBr + Nerolidol	5 µg/mL ± 0.5 *
Norfloxacin + Farnesol	64 µg/mL ± 0.5	EtBr + Farnesol	50.8 µg/mL ± 0.8 *
Norfloxacin + α-bisabolol	64 µg/mL ± 0.5	EtBr + α-bisabolol	80.6 µg/mL ± 0.8
Norfloxacin + Liposome control	101.6 µg/mL ± 0.8	EtBr + Liposome control	64 µg/mL ± 0.5
Norfloxacin + Liposome/Nerolidol	64 µg/mL ± 0.5	EtBr + Liposome/Nerolidol	64 µg/mL ± 0.5
Norfloxacin + Liposome/Farnesol	101.6 µg/mL ± 0.8	EtBr + Liposome/Farnesol	40.3 µg/mL ± 0.8 *
Norfloxacin + Liposome/α-bisabolol	64 µg/mL ± 0.5	EtBr + Liposome/α-bisabolol	50.8 µg/mL ± 0.8 *

**Table 4:** Minimum inhibitory concentration of nerolidol, farnesol, and α-bisabolol sesquiterpenes, whether isolated or encapsulated in liposomes, against the *S. aureus* IS-58 strain, associated with norfloxacin and ethidium bromide. * Statistically significant compared to the tetracyclin or EtBr control.

Group with Antibiotic	MIC	Group with EtBr	MIC
Tetracycline	64 µg/mL ± 0.5	EtBr	10 µg/mL ± 0.5
Tetracycline + CCCP	25.4 µg/mL ± 0.8 *	EtBr + CCCP	2 µg/mL ± 0.5 *
Tetracycline + Nerolidol	64 µg/mL ± 0.5	EtBr + Nerolidol	25.4 µg/mL ± 0.8
Tetracycline + Farnesol	64 µg/mL ± 0.5	EtBr + Farnesol	10 µg/mL ± 0.5
Tetracycline + α-bisabolol	64 µg/mL ± 0.5	EtBr + α-bisabolol	8 µg/mL ± 0.5 *
Tetracycline + Liposome control	128 µg/mL ± 0.5	EtBr + Liposome control	36 µg/mL ± 0.5
Tetracycline + Liposome/Nerolidol	256 µg/mL ± 0.5	EtBr + Liposome/Nerolidol	36 µg/mL ± 0.5
Tetracycline + Liposome/Farnesol	128 µg/mL ± 0.5	EtBr + Liposome/Farnesol	36 µg/mL ± 0.5
Tetracycline + Liposome/α-bisabolol	64 µg/mL ± 0.5	EtBr + Liposome/α-bisabolol	12.7 µg/mL ± 0.8

**Table 5:** Minimum inhibitory concentration of nerolidol, farnesol, and α-bisabolol sesquiterpenes, whether isolated or encapsulated in liposomes, against the *S. aureus* RN4220 strain, associated with norfloxacin and ethidium bromide. * Statistically significant compared to the tetracyclin or EtBr control.

Group with Antibiotic	MIC	Group with EtBr	MIC
Erythromycin	512 µg/mL ± 0.5	EtBr	36 µg/mL ± 0.5
Erythromycin + CCCP	0.5 µg/mL ± 0.5 *	EtBr + CCCP	2 µg/mL ± 0.5 *
Erythromycin + Nerolidol	512 µg/mL ± 0.5	EtBr + Nerolidol	36 µg/mL ± 0.5
Erythromycin + Farnesol	256 µg/mL ± 0.5 *	EtBr + Farnesol	21.8 µg/mL ± 0.8 *
Erythromycin + α-bisabolol	512 µg/mL ± 0.5	EtBr + α-bisabolol	36 µg/mL ± 0.5
Erythromycin + Liposome control	512 µg/mL ± 0.5	EtBr + Liposome control	36 µg/mL ± 0.5
Erythromycin + Liposome/Nerolidol	512 µg/mL ± 0.5	EtBr + Liposome/Nerolidol	8 µg/mL ± 0.5 *
Erythromycin + Liposome/Farnesol	512 µg/mL ± 0.5	EtBr + Liposome/Farnesol	21.8 µg/mL ± 0.8 *
Erythromycin + Liposome/α-bisabolol	512 µg/mL ± 0.5	EtBr + Liposome/α-bisabolol	36 µg/mL ± 0.5

**Table 6:** Bacterial strains of *S. aureus* used in the microbiological assays.

Strain	Plasmid/Gene	Protein (Substrate Antibiotic)
1199B	*norA*	NorA (Norfloxacin)
IS-58	Plasmid PT181 (*tetK*)	Tet(K) (Tetracyclin)
RN4220	Plasmid Pul5054 (*msrA*)	MsrA (Erythromycin)

## Data Availability

All experimental data generated or analyzed during this study are included in the article.
